# Red squirrels exhibit antipredator behavioural changes in response to a native predator, the pine marten

**DOI:** 10.1098/rsos.250661

**Published:** 2025-06-18

**Authors:** Emily Reilly, Colin Lawton

**Affiliations:** ^1^ Department of Zoology, University of Galway, Galway, Ireland

**Keywords:** behaviour, *Martes martes*, predator detection, *Sciurus vulgaris*, trail camera, vigilance

## Abstract

Prey that coevolve alongside their predators develop specific antipredator responses to reduce their predation risk. Red squirrels (*Sciurus vulgaris*) are one such prey species who share an evolutionary history with a predator, the pine marten (*Martes martes*). The recent resurgence of the pine marten has caused a decline in the invasive grey squirrel (*Sciurus carolinensis*) in Ireland; however, it has not had the same impact on the Irish red squirrel population. We used trail cameras to record pine marten and red squirrel visits to feeders and analysed the behaviour of the red squirrel following recent pine marten presence. We found that red squirrels displayed an enhanced antipredator response involving increased vigilance, and decreased feeding following a visit from a pine marten. This effect was strongest with increasing proximity to the pine marten visit and weakened over time. These results indicate that red squirrels can detect recent pine marten presence and assess the perceived risk of predation based on the time since the predator’s visit. These behavioural adaptations and sensitivity to the recent presence of the pine marten are hypothesized to have allowed for the red squirrel population recovery, in direct contrast to the grey squirrel decline in Ireland.

## 1. Introduction

Animals in the wild face constant danger from a wide range of threats. One of the biggest threats an animal faces is that of a predator. Antipredator adaptations are traits that evolve through natural selection in the prey species to assist in their defence against a predator [1,2]. Some of these adaptations result in permanent antipredator responses that include but are not limited to changes in morphology [3], physiology [4] and life history [5,6]. Antipredator adaptations may also be expressed temporarily, such as changes in behaviour [7]. Plasticity in antipredator traits allows the animal to minimize the costs of expressing this trait by not employing them when there is a low risk of predation [8]. The recent presence of a predator can trigger a predator response sequence that allows for the expression of an antipredator trait [9]. First, the prey detects the recent presence of the predator, then it recognizes the threat and assesses the level of danger, and lastly, it displays the appropriate antipredator response. If the prey fails in any of these steps, it will be at a greater risk of predation.

The first step in the antipredator response sequence is to detect the presence of a predator. Prey that experience a greater proportional fitness loss if attacked will detect the predation risk earlier than prey who would experience a lower proportional fitness loss [10]. Predator detection is often facilitated by the cue of a predator. Cues may be chemical [11], visual [12], mechanical [13–15] and/or auditory [16–18]. Chemical cues include odours left by a predator, often through sources such as skin, fur, faeces, urine and gland secretions [19]. These olfactory cues may be left inadvertently by the predator or deliberately for communication [20]. Olfactory cues are especially important for mammalian prey who rely heavily on scent detection and identification in other activities such as foraging and communication [21,22]. Olfactory cues may also be left without physical secretion by the predator [19]. The predator may indirectly transfer their scent while interacting with their environment through physical contact alone, such as by rubbing against surfaces. It has been hypothesized that such cues may illicit a greater antipredator response compared to feces or urine-derived cues, because they represent a more reliable indication of imminent predator presence [23].

Once the prey has detected the recent presence of the predator, it must then recognize the potential threat and assess the danger of the situation. The recognition of a predator can be both learned [9,20,24] and inherited as a result of coevolution [2,25–28]. The level of risk can be determined using additional information revealed by the cue. Not only does the cue reveal the location of the predator at an earlier point in time, but the age or ‘freshness’ can reveal how long ago it was left by the predator [29]. Cues can also reveal the direction in which the potential predator was travelling [30]. All of this information can be used by the prey to assess the likelihood of predation and react accordingly. The subsequent reaction to the predator’s recent presence is the final stage of the antipredator response sequence [9].

The detection of a predator generates a trade-off for the prey whereby the prey must weigh the cost of the perceived risk of predation against the benefits of opportunities such as mating or feeding [31]. This trade-off is often expressed as a behavioural change in the prey animal. Behavioural responses are specific to the prey–predator relationship but generally involve reduced activity [32–34], increased refuge use (i.e. hiding [35]), fleeing [11,36,37] and altering their habitat use [38,39]. For olfactory cues, the behavioural change will also vary depending on the freshness of the scent. Prey have been shown to reduce foraging behaviour when the predator’s scent is fresh, increase it as the predation risk drops, and stop responding once the cue has aged [29,40].

The red squirrel and the pine marten are both native to Ireland and share an evolutionary history [41–44]. A simulation of pine marten presence has been shown to affect the behaviour of the red squirrel [45]. Red squirrels reacted to the cue by reducing the number of visits to the feeder, reducing the time spent feeding and increasing the time spent vigilant. Conversely, the invasive grey squirrel (*Sciurus carolinensis*) shows a lack of an antipredator response in the presence of a pine marten cue [46]. This predator naivety, coupled with the resurgence of the pine marten, has led to the decline of the grey squirrel population in Ireland [47–50]. Interestingly, the recovery of the pine marten has not had the same impact on red squirrel populations, despite it being the natural predator of this species [47]. Sheehy *et al*. [50] have hypothesized that this coexistence is possible because the red squirrel is able to avoid the pine marten due to its inherent antipredator response, which the grey squirrel lacks. Understanding the pine marten and grey squirrel dynamic is particularly important given the severely detrimental impact of the grey squirrel on red squirrel populations in Europe, which has resulted in local extinctions and significant population declines [51].

The aim of this project was to investigate and analyse the antipredator behavioural adaptations of red squirrels in response to recent pine marten presence. While Twining *et al*. [45] simulated the presence of pine marten by using a scat/water mixture, this study sought to investigate whether red squirrels could detect and react to the recent presence of a pine marten. Trail cameras were used to record red squirrel and pine marten visits to a feeder, and subsequently, the behavioural changes of red squirrels were analysed. It was hypothesized that red squirrels would detect the recent presence of a pine marten and display antipredator responses such as decreased activity, decreased feeding and increased vigilance. Additionally, it was hypothesized that these responses would become less pronounced with time since the pine marten visit, until normal behaviour was resumed.

## 2. Methodology

Data were collected from January 2020 to May 2021 at two sites in the west of Ireland. Red squirrels and pine martens were present at both sites. Derryclare woods (570 ha) in Connemara is a commercial forest consisting mainly of lodgepole pine (*Pinus contorta*) and Sitka spruce (*Picea sitchensis*) stands. It also contains a small (12 ha) broadleaf stand, consisting mainly of oak (*Quercus petraea*), ash (*Fraxinus excelsior*), birch (*Betula* sp.) and hazel (*Corylus avellana*). The second site, Belleek Woods in Ballina, Co. Mayo (61 ha), is a public park with a mix of broadleaf (mainly beech, *Fagus sylvatica*) and conifer trees (mainly Norway spruce, *Picea abies*). Sampling in Belleek lasted from February 2020 to September 2020 and Derryclare data were collected from January 2020 to May 2021. The number of videos recorded in Derryclare was much lower than in Belleek, so the duration of the study was extended at that site.

Bushnell NatureView HD and Browning trail cameras were used to capture pine marten and red squirrel visits to feeders. Squirrels were considered to have 'visited' the feeder if they approached within approximately one meter of it, with or without touching it. All pine marten visits captured involved an interaction with the feeder. The video length was set at 30 s, in order to capture either pine marten presence, or the presence and initial response of the squirrel. It was not possible to identify either squirrels or pine marten to the level of individuals due to a lack of visual identifiers. Two designs of feeders were used: hairtubes and feeding stations. Hairtubes consisted of 30 cm long 65 × 65 mm square PVC pipes secured to trees between chest and head height. The feeding stations consisted of a rectangular box (L20 cm × W15 cm × H15 cm) with a hinged lid and a small platform on which the animal could stand. Bait for the feeders consisted of hazelnuts and peanuts, and was refilled intermittently from once every few days to once every two months. Four cameras were deployed in each site, at least 150 m apart, giving a total of eight cameras. Trail cameras were secured to a tree within 6 m of the feeder. During every visit to a site the camera batteries and memory cards were changed. In Belleek, cameras were in place for a total of seven months, at a density of one camera per 15 ha of mature woodland. Cameras were positioned at their feeder for an average of 210 ± 65 days/feeder. In Derryclare, cameras were in place for 17 months, at a density of one camera per 50.5 ha of mature woodland. All cameras in Derryclare were relocated to a different feeder halfway through the study, in order to maximize the range and number of individuals from both species monitored. Cameras were positioned at a feeder for an average of 255 ± 51 days per feeder.

Behaviour was analysed using BORIS behavioural software [52]. For red squirrels, we recorded and analysed the following variables: the hour of the day in which the visit occurred (e.g. 15.00), whether the visit occurred during the squirrel breeding season (February–July [53]), the visit duration (i.e. the length of time the squirrel spent in frame during the video), whether or not bait was available, the number of minutes since the most recent pine marten visit, referred to as 'time since marten', and the number of seconds the squirrel spent (i) feeding, (ii) showing vigilance and (iii) sniffing.

Red squirrels usually feed in a hunched posture, with the squirrel using its front limbs to hold the food to its mouth, while chewing. At times red squirrels feed in a quadrupedal stance with the head lowered to the food, also while chewing. Sniffing was normally conducted by the red squirrels with their heads lowered to the feeder. Vigilance was recorded when the red squirrels were completely immobile—usually sitting or standing—with the head raised and alert, or any time the red squirrel was immobile but flicking its tail. This behaviour, known as tail flagging, is considered to be an alarm response [54].

Both the visit durations and behavioural factors (feeding, vigilance and sniffing) were measured in seconds. The residuals were tested for normality, and all were found to not have a normal distribution. The duration of vigilance, sniffing and feeding were transformed by adding a constant of 0.1 before applying a log_10_ transformation to meet the assumptions of normality. Visit duration was also log_10_-transformed.

Generalized linear models (GLMs) with Type III sum-of-squares analysis were conducted to determine if the pine marten visit affected the duration of squirrel visits, and the occurrence of vigilant, sniffing and feeding behaviour. Fixed effects included the time of the visit and whether it occurred during the red squirrel breeding season. Time since marten was log_10_-transformed and used as a covariate. Furthermore, the effect of a pine marten visit on feeding behaviour during squirrel visits within the subsequent 8 h was analysed using a *t*‐test.

The visit durations and the behaviours of sniffing and feeding were tested only for visits during which bait was available, in order to control for food availability as a factor.

Analyses were performed using IBM SPSS Statistics 27.

## 3. Results

A total of 491 squirrel visits to feeders were recorded (Belleek = 454, Derryclare = 37), with a total of 7874 s of red squirrel presence. A total of 226 pine marten visits were recorded (Belleek = 81, Derryclare = 145). Bait was visible for a total of 202 squirrel visits, and this subset was used in analyses of visit duration, and sniffing and feeding behaviours.

**Figure 1.**
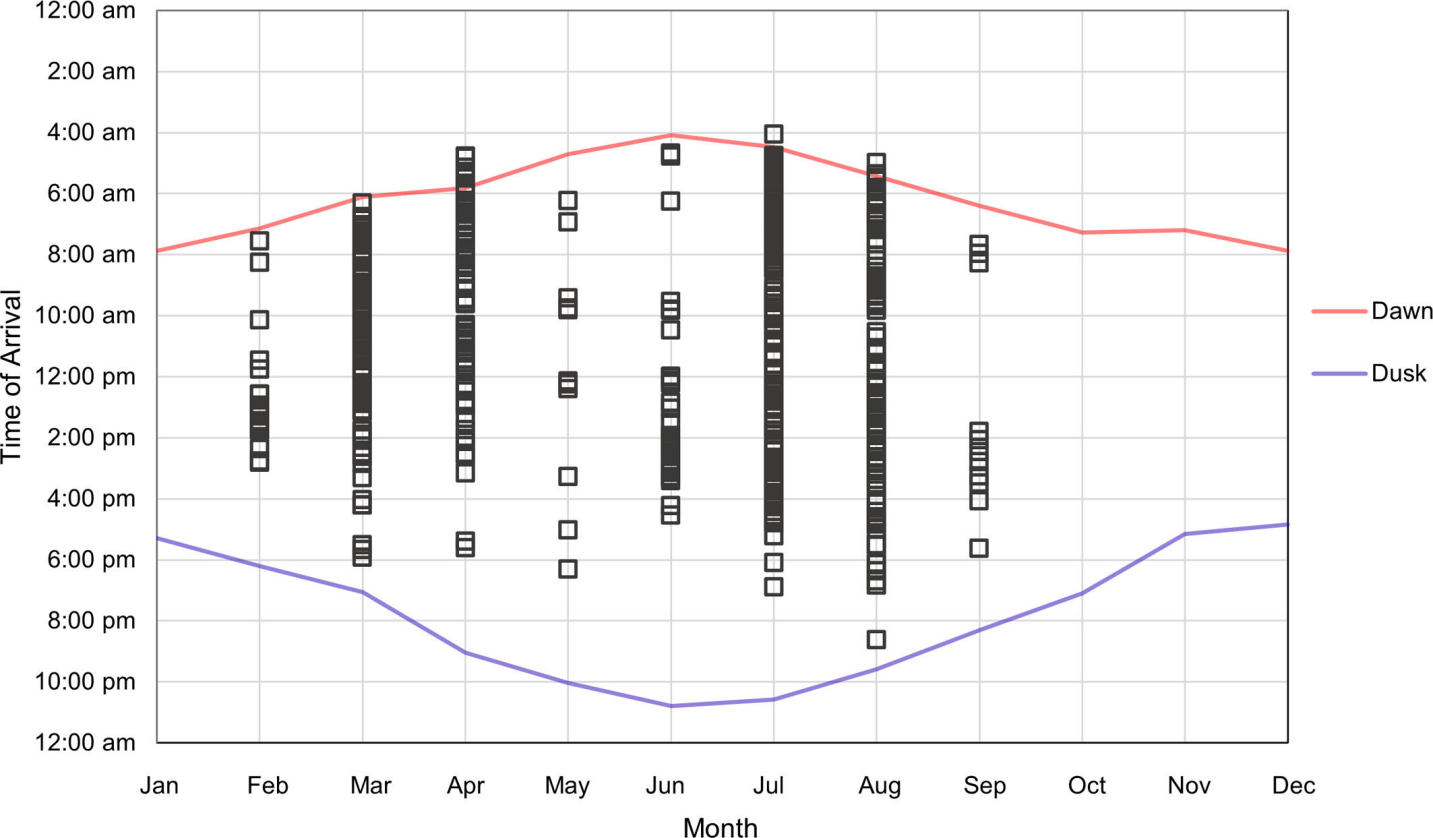
Red squirrel arrival times recorded at a feeder in the months of February–September.

**Figure 2.**
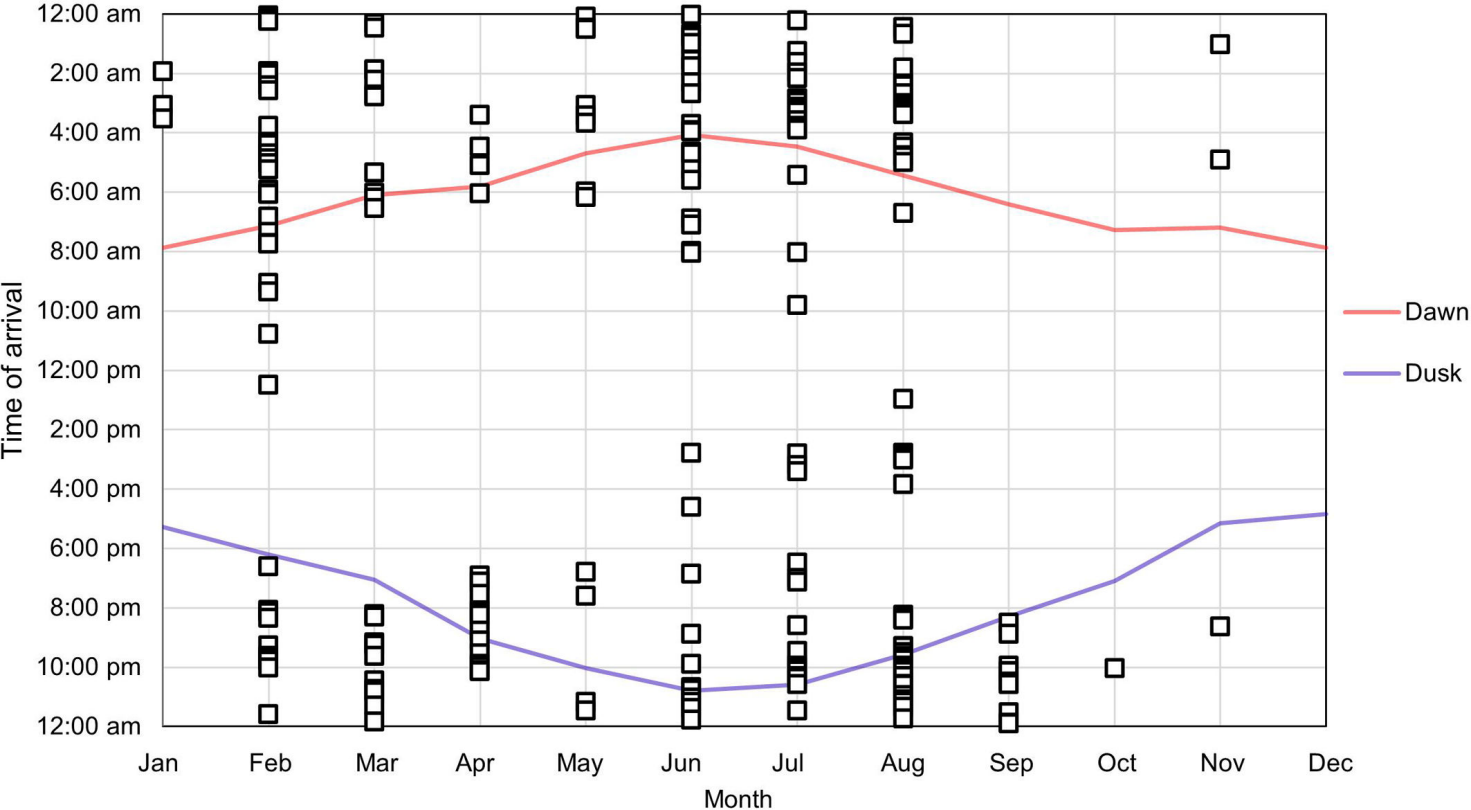
Pine marten recorded arrival times at a feeder in the months of January–November.

Squirrels usually visited the feeders from dawn to just before dusk, with the earliest and latest recordings occurring in the summer months. Pine marten mostly visited the feeders at night, but also occasionally during daylight hours. Pine marten activity overlapped with squirrel activity in the mornings for all months, but this overlap is more pronounced in some months, with some pine marten recordings occurring as late in the day as 12.58 (see figures 1 and 2).

The duration of squirrel visits was not significantly affected by the recent presence of the pine marten (*F* (1, 202) = 1.257, *p* = 0.264) (table 1). Whether the visit occurred during breeding season had a significant effect on the duration of visits, with squirrels in the breeding season making longer visits than squirrels in the non-breeding season (*F* (1, 202) = 4.027, *p* = 0.046). The time of the visit also significantly affected the duration of visits (*F* (14, 189) = 2.260, *p* = 0.007), although no clear pattern emerged.

**Table 1.** A summary of the GLMs performed on the four behavioural variables: visit duration, sniffing, vigilance and feeding. Significant results are shown in bold.

	visit duration (*n* = 202)					sniffing (*n* = 202)					vigilance (*n* = 491)					feeding (*n* = 202)				
	*B*	s.e.	*df*	*F*	*p*	*B*	s.e.	*df*	*F*	*p*	*B*	s.e.	*df*	*F*	*p*	*B*	s.e.	*df*	*F*	*p*
intercept	0.991	0.228	1	**296.017**	**<0.001**	1.031	0.940	1	2.607	0.108	0.257	0.865	1	**27.689**	**<0.001**	−1.963	1.006	1	**7.061**	**0.009**
time since marten	−0.022	0.020	1	1.257	0.264	−0.104	0.082	1	1.606	0.207	−0.308	0.041	1	**56.951**	**<0.001**	0.195	0.088	1	**4.948**	**0.027**
breeding season (no)	−0.189	0.216	1	**4.027**	**0.046**	0.853	0.889	1	2.952	0.087	−0.259	0.501	1	3.820	0.051	−0.653	0.307	1	**4.526**	**0.035**
time of visit	—	—	14	2.260	0.007	—	—	14	0.937	0.520	—	—	14	0.798	0.68	—	—	14	1.159	0.310

Sniffing was similarly unaffected by the recent presence of the pine marten (*F* (1, 202) = 1.606, *p* = 0.207). Additionally, breeding season (*p* = 0.087) and the hour of arrival (*p* = 0.520) did not significantly affect sniffing behaviour.

Vigilance was significantly negatively affected by the recent presence of the pine marten, meaning that the time squirrels spent being vigilant decreased as the time since the pine marten’s visit increased (*B* = −0.308, s.e. = 0.041, *F* (1, 489) = 56.951, *p* < 0.001). Whether or not the visit occurred during the breeding season had no effect on vigilance (*F* (1, 489) = 3.820, *p =* 0.051), nor did the time of the squirrel’s visit (F (1, 489) = 0.798, *p =* 0.680).

**Figure 3.**
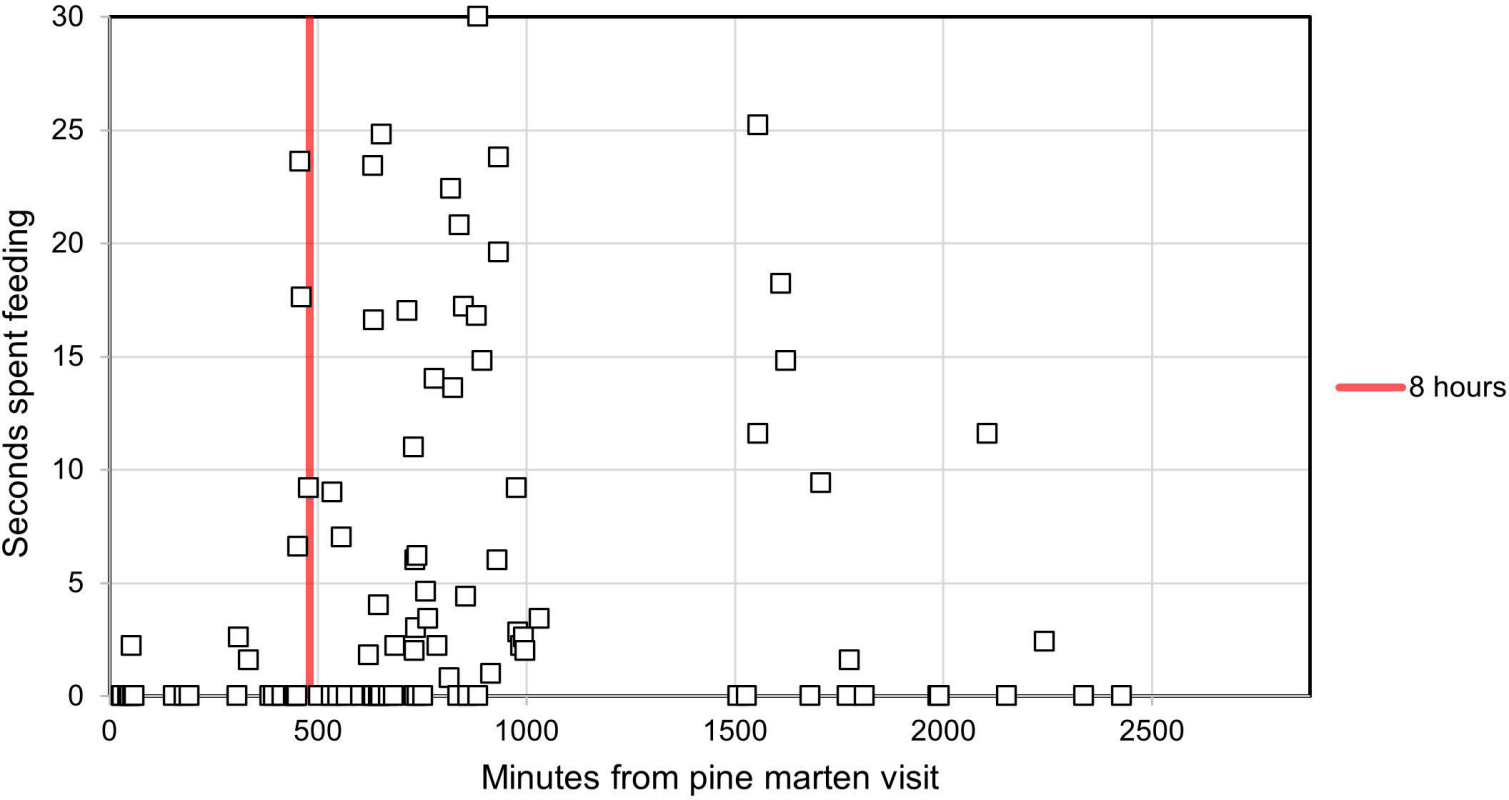
A closer look at the initial 48 h following a pine marten visit, showing the number of seconds each red squirrel spent feeding on visits when bait was available, plotted against the number of minutes since the most recent pine marten visit. Squirrels spent significantly less time feeding in the first 8 h following a pine marten visit.

Feeding was significantly positively affected by the recent presence of the pine marten (*B* = 0.195, s.e. = 0.088, *F* (1, 202) = 4.948, *p* = 0.027). While feeding behaviour increased significantly with increasing time from the pine marten visit, the data also revealed a significant lack of feeding in the initial 8 hours following a pine marten’s presence (*t* (199) = −2.876, *p* = 0.004) (figure 3).

Squirrels who visited during the non-breeding season spent significantly less time feeding compared to those who visited in the breeding season (*F* (14, 189) = 4.526, *p* = 0.035). The time of the visit did not significantly affect feeding duration (*F* (1, 202) = 1.159, *p* = 0.310).

## 4. Discussion

In this study, we have shown that red squirrels alter their behaviour in response to recent pine marten presence. Following a pine marten visit to a feeder, red squirrels displayed a typical antipredator response, exhibiting increased vigilance and decreased feeding. There was no difference in the duration of the red squirrels’ visits to a feeder, meaning red squirrels did not display a flight reaction in response to recent pine marten presence.

Red squirrel and pine marten activity patterns overlapped during daylight hours in some months, particularly in the summer (figures 1 and 2). It was found that the pine marten was active during daylight hours for some of the year, which is in line with the literature [55,56].

The first two stages in the antipredator response sequence involve detection, followed by recognition and assessment [9]. Upon recognizing the scent of a predator, the prey will display an antipredator reaction, such as fleeing [11,36,37]. However, for visits when bait was present, the length of the visit was not significantly affected by the number of minutes since the pine marten’s visit. Recent pine marten presence was predicted to elicit a flight response from the red squirrels, resulting in the visits immediately following a pine marten visit being the shortest, in keeping with the literature [45]. However, this was not the case. A flight response, if it were to occur, would be expected to happen immediately after the threat is recognized [57]. Therefore, a video length of 30 s is adequate to record a flight response. The absence of a flight response indicates that the red squirrels chose continued foraging over fleeing. This may be because the perceived predation risk is low, based on the strength of the cue [58]. Another possible response following the recognition of a predator’s cue is avoidance [11,19,39]. However, the number of red squirrel visits recorded following a pine marten visit suggests that red squirrels did not show an avoidance response.

In the absence of a flight response, other common antipredator responses such as a trade-off between increased vigilance and decreased feeding may be expressed [26,59–61]. Following a pine marten visit, red squirrels spent more time being vigilant at the feeder. The inverse was seen in feeding behaviour. Squirrels fed less following a pine marten visit, and increased their feeding behaviour with increasing time from the pine marten’s presence. These results show that red squirrels do display an antipredator reaction in response to recent pine marten presence, in the form of increased vigilance and decreased feeding, rather than flight.

The effect of a predator’s presence on antipredator behaviours diminishes over time as the cue ages and the perceived risk of predation decreases [29,62]. This diminishment can be seen clearly in the decrease in vigilance, and the increase in feeding, as indicated by the regression coefficient (*B*). The starkest response to the recent presence of the pine marten was seen in feeding behaviour. Red squirrels fed extremely rarely in the 8 h following a pine marten visit, despite the availability of bait (figure 3). The subsequent weakening of the red squirrel antipredator response with increasing time from pine marten presence is in line with previous research [45]. The current study’s results indicate that red squirrels can evaluate the cost-benefit relationship of displaying antipredator responses and react accordingly.

The grey squirrel does not have a shared evolutionary history with the pine marten and has shown an inability to recognize the danger associated with the cue of the pine marten and display an antipredator response [45]. This is hypothesized to be one of the factors contributing to the grey squirrel’s decline in Ireland and Scotland [45,50]. The antipredator behaviours displayed by the red squirrel in this study are probably a product of their coevolution with the pine marten. Recent pine marten presence triggers a response where they become more vigilant and reduce their feeding activity. In doing so, they allocate more time to investigate the perceived threat, thereby reducing the risk of predation. These data support the hypothesis that the red squirrel’s antipredator response has allowed for the red squirrel population recovery, despite the resurgence of their native predator, in direct contrast to the grey squirrel decline [45,50]. Additionally, these data demonstrate that red squirrels can detect recent pine marten presence, in the absence of defecating or urinating. The red squirrel’s behavioural adaptations and sensitivity to the recent presence of the pine marten help to explain the conflicting outcomes of two squirrel species in the presence of a shared predator.

## Data Availability

Data are available at Zenodo [63]. SPSS was used for analyses and therefore we have no code.

## References

[1] Aguiar O, Sonnega S, DiNuzzo ER, Sheriff MJ. 2023 Playing it safe; risk‐induced trait responses increase survival in the face of predation. J. Anim. Ecol. **92**, 690–697. (10.1111/1365-2656.13880) 36597705

[2] Dugatkin LA. 2008 Antipredation behavior. In Encyclopedia of ecology (ed. BD Fath), pp. 218–221. Annapolis, MD, USA: Elsevier. (10.1016/B978-008045405-4.00004-5)

[3] Cairns J, Moerman F, Fronhofer EA, Altermatt F, Hiltunen T. 2020 Evolution in interacting species alters predator life-history traits, behaviour and morphology in experimental microbial communities. Proc. R. Soc. B Biol. Sci. **287**, 20200652. (10.1098/rspb.2020.0652) PMC7341940 32486984

[4] Ruxton GD, Allen WL, Sherratt TN, Speed MP. 2019 Avoiding attack: the evolutionary ecology of crypsis, aposematism, and mimicry, pp. 84–102. Oxford: Oxford University Press. (10.1093/oso/9780199688678.003.0007)

[5] Brodersen J, Howeth JG, Post DM. 2015 Emergence of a novel prey life history promotes contemporary sympatric diversification in a top predator. Nat. Commun. **6**, 8115. (10.1038/ncomms9115) 26365323

[6] Sih A, Bolnick DI, Luttbeg B, Orrock JL, Peacor SD, Pintor LM, Preisser E, Rehage JS, Vonesh JR. 2010 Predator-prey naïveté, antipredator behavior, and the ecology of predator invasions. Oikos **119**, 610–621. (10.1111/j.1600-0706.2009.18039.x)

[7] Barnard C. 2012 Animal behaviour: ecology and evolution. Heidelberg, Germany: Springer Science & Business Media. (10.1007/978-1-4615-9781-0)

[8] Agrawal AA. 2001 Phenotypic plasticity in the interactions and evolution of species. Science **294**, 321–326. (10.1126/science.1060701) 11598291

[9] Kelley JL, Magurran AE. 2003 Learned predator recognition and antipredator responses in fishes. Fish Fish. **4**, 216–226. (10.1046/j.1467-2979.2003.00126.x)

[10] Sheriff MJ, Orrock JL, Ferrari MCO, Karban R, Preisser EL, Sih A, Thaler JS. 2020 Proportional fitness loss and the timing of defensive investment: a cohesive framework across animals and plants. Oecologia **193**, 273–283. (10.1007/s00442-020-04681-1) 32542471

[11] Kats LB, Dill LM. 1998 The scent of death: chemosensory assessment of predation risk by prey animals. Écoscience **5**, 361–394. (10.1080/11956860.1998.11682468)

[12] Blumstein DT, Daniel JC, Griffin AS, Evans CS. 2000 Insular tammar wallabies (Macropus eugenii) respond to visual but not acoustic cues from predators. Behav. Ecol. **11**, 528–535. (10.1093/beheco/11.5.528)

[13] Castellanos I, Barbosa P. 2006 Evaluation of predation risk by a caterpillar using substrate-borne vibrations. Anim. Behav. **72**, 461–469. (10.1016/j.anbehav.2006.02.005)

[14] Djemai I, Casas J, Magal C. 2001 Matching host reactions to parasitoid wasp vibrations. Proc. R. Soc. B Biol. Sci. **268**, 2403–2408. (10.1098/rspb.2001.1811) PMC1088893 11747557

[15] Virant-Doberlet M, Kuhelj A, Polajnar J, Šturm R. 2019 Predator-prey interactions and eavesdropping in vibrational communication networks. Front. Ecol. Evol. **7**, 203. (10.3389/fevo.2019.00203)

[16] Pollack GS. 2016 Hearing for defense. In Insect hearing (eds GS Pollack, AC Mason, AN Popper, RR Fay), pp. 81–98. Cham, Switzerland: Springer International Publishing. (10.1007/978-3-319-28890-1_4)

[17] Yack JE, Raven BH, Leveillee MB, Naranjo M. 2020 What does an insect hear? Reassessing the role of hearing in predator avoidance with insights from vertebrate prey. Integr. Comp. Biol. **60**, 1036–1057. (10.1093/icb/icaa097) 32717080

[18] Jayne K, Lea SEG, Leaver LA. 2015 Behavioural responses of eastern grey squirrels, Sciurus carolinensis, to cues of risk while foraging. Behav. Process. **116**, 53–61. (10.1016/j.beproc.2015.05.002) 25957953

[19] Apfelbach R, Blanchard CD, Blanchard RJ, Hayes RA, McGregor IS. 2005 The effects of predator odors in mammalian prey species: a review of field and laboratory studies. Neurosci. Biobehav. Rev. **29**, 1123–1144. (10.1016/j.neubiorev.2005.05.005) 16085312

[20] Wyatt TD. 2010 Pheromones and signature mixtures: defining species-wide signals and variable cues for identity in both invertebrates and vertebrates. J. Comp. Physiol. **196**, 685–700. (10.1007/s00359-010-0564-y) 20680632

[21] Banks PB, Bytheway JP, Carthey A, Hughes NK, Price CJ. 2014 Olfaction and predator-prey interactions amongst mammals in Australia. In Carnivores of australia: past, present and future (ed. AS Glen CRD), pp. 389–404. Collingwood, Australia: CSIRO Publishing.

[22] Lledo PM, Gheusi G, Vincent JD. 2005 Information processing in the mammalian olfactory system. Physiol. Rev. **85**, 281–317. (10.1152/physrev.00008.2004) 15618482

[23] Blanchard DC, Griebel G, Blanchard RJ. 2003 Conditioning and residual emotionality effects of predator stimuli: some reflections on stress and emotion. Prog. Neuro Psychopharmacol. Biol. Psychiatry **27**, 1177–1185. (10.1016/j.pnpbp.2003.09.012) 14659473

[24] Hudson CM, Brown GP, Shine R. 2017 Evolutionary shifts in anti-predator responses of invasive cane toads (Rhinella marina). Behav. Ecol. Sociobiol. **71**, 134. (10.1007/s00265-017-2367-4)

[25] Binz H, Bucher R, Entling MH, Menzel F. 2014 Knowing the risk: crickets distinguish between spider predators of different size and commonness. Ethology **120**, 99–110. (10.1111/eth.12183)

[26] Carthey AJR, Banks PB. 2016 Naiveté is not forever: responses of a vulnerable native rodent to its long term alien predators. Oikos **125**, 918–926. (10.1111/oik.02723)

[27] Kempraj V, Park SJ, Taylor PW. 2020 Forewarned is forearmed: Queensland fruit flies detect olfactory cues from predators and respond with predator-specific behaviour. Sci. Rep. **10**, 7297. (10.1038/s41598-020-64138-6) 32350381 PMC7190731

[28] Tariel J, Plénet S, Luquet É. 2020 Transgenerational plasticity in the context of predator-prey interactions. Front. Ecol. Evol. **8**, 548660. (10.3389/fevo.2020.548660)

[29] Bytheway JP, Carthey AJR, Banks PB. 2013 Risk vs. reward: how predators and prey respond to aging olfactory cues. Behav. Ecol. Sociobiol. **67**, 715–725. (10.1007/s00265-013-1494-9)

[30] Steck K. 2012 Just follow your nose: homing by olfactory cues in ants. Curr. Opin. Neurobiol. **22**, 231–235. (10.1016/j.conb.2011.10.011) 22137100

[31] Siepielski AM, Fallon E, Boersma K. 2016 Predator olfactory cues generate a foraging–predation trade-off through prey apprehension. R. Soc. Open Sci. **3**, 150537. (10.1098/rsos.150537) 26998324 PMC4785975

[32] Siegal E, Hooker SK, Isojunno S, Miller PJO. 2022 Beaked whales and state-dependent decision-making: how does body condition affect the trade-off between foraging and predator avoidance? Proc. R. Soc. B **289**, 20212539. (10.1098/rspb.2021.2539) PMC8790365 35078370

[33] Van Buskirk J, Yurewicz K. 1998 Effects of predators on prey growth rate: relative contributions of thinning and reduced activity. Oikos **82**, 20–28.

[34] Van Duren L, Videler J. 1996 The trade-off between feeding, mate seeking and predator avoidance in copepods: behavioural responses to chemical cues. J. Plankton Res. **18**, 805–818.

[35] Stauffer HP, Semlitsch RD. 1993 Effects of visual, chemical and tactile cues of fish on the behavioural responses of tadpoles. Anim. Behav. **46**, 355–364. (10.1006/anbe.1993.1197)

[36] Lingle S, Pellis S. 2002 Fight or flight? Antipredator behavior and the escalation of coyote encounters with deer. Oecologia **131**, 154–164. (10.1007/s00442-001-0858-4) 28547505

[37] Randler C. 2006 Red squirrels (Sciurus vulgaris) respond to alarm calls of Eurasian jays (Garrulus glandarius). Ethology **112**, 411–416. (10.1111/j.1439-0310.2006.01191.x)

[38] Dickman C. 1992 Predation and habitat shift in the house mouse, Mus domesticus. Ecology **73**, 313–322.

[39] Stoddart D. 1982 Does trap odour influence estimation of population size of the short-tailed vole, Microtus agrestis? J. Anim. Ecol **51**, 375–386.

[40] Koivisto E, Pusenius J. 2003 Effects of temporal variation in the risk of predation by least weasel (Mustela nivalis) on feeding behavior of field vole (Microtus agrestis). Evol. Ecol. **17**, 477–489. (10.1023/B:EVEC.0000005594.40721.17)

[41] Moffat CB. 1937 The mammals of Ireland. Proc. R. Ir. Acad. B. **44**, 61–128.

[42] Montgomery WI, Provan J, McCabe AM, Yalden DW. 2014 Origin of British and Irish mammals: disparate post-glacial colonisation and species introductions. Quat. Sci. Rev. **98**, 144–165. (10.1016/j.quascirev.2014.05.026)

[43] O’sullivan PJ. 1983 The distribution of the pine marten (Martes martes) in the Republic of Ireland. Mammal Rev. **13**, 39–44. (10.1111/j.1365-2907.1983.tb00265.x)

[44] O’Mahony D, O’Reilly C, Turner P. 2006 National pine marten survey of Ireland 2005. COFORD Connect. Environment. **7**, 1–8.

[45] Twining JP, Ian Montgomery W, Price L, Kunc HP, Tosh DG. 2020 Native and invasive squirrels show different behavioural responses to scent of a shared native predator. R. Soc. Open Sci. **7**, 191841. (10.1098/rsos.191841) 32257340 PMC7062111

[46] Twining JP, Lawton C, White A, Sheehy E, Hobson K, Montgomery WI, Lambin X. 2022 Restoring vertebrate predator populations can provide landscape‐scale biological control of established invasive vertebrates: insights from pine marten recovery in Europe. Glob. Chang. Biol. **28**, 5368–5384. (10.1111/gcb.16236) 35706099 PMC9542606

[47] Lawton C, Flaherty M, Goldstein E, Sheehy E, Carey M. 2015 Irish squirrel survey 2012. Dublin, Ireland: Irish Wildlife Manuals.

[48] Lawton C, Hanniffy R, Molloy V, Guilfoyle C, Stinson M, Reilly E. 2019 All-Ireland squirrel and pine marten survey 2019. Dublin, Ireland: Irish Wildlife Manuals.

[49] Sheehy E, Lawton C. 2014 Population crash in an invasive species following the recovery of a native predator: the case of the American grey squirrel and the European pine marten in Ireland. Biodivers. Conserv. **23**, 753–774. (10.1007/s10531-014-0632-7)

[50] Sheehy E, Sutherland C, O’Reilly C, Lambin X. 2018 The enemy of my enemy is my friend: native pine marten recovery reverses the decline of the red squirrel by suppressing grey squirrel populations. Proc. R. Soc. B Biol. Sci. **285**, 20172603. (10.1098/rspb.2017.2603) PMC5879625 29514972

[51] Wauters LA, Lurz PWW, Santicchia F, Romeo C, Ferrari N, Martinoli A, Gurnell J. 2023 Interactions between native and invasive species: a systematic review of the red squirrel-gray squirrel paradigm. Front. Ecol. Evol. **11**, 1083008. (10.3389/fevo.2023.1083008)

[52] Friard O, Gamba M. 2016 BORIS: a free, versatile open‐source event‐logging software for video/audio coding and live observations. Methods Ecol. Evol. **7**, 1325–1330. (10.1111/2041-210x.12584)

[53] Lurz PW, Gurnell J, Magris L. 2005 Sciurus vulgaris. Mamm. Species **769**, 1–10. (10.1644/1545-1410(2005)769[0001:SV]2.0.CO;2)

[54] Partan SR, Larco CP, Owens MJ. 2009 Wild tree squirrels respond with multisensory enhancement to conspecific robot alarm behaviour. Anim. Behav. **77**, 1127–1135. (10.1016/j.anbehav.2008.12.029)

[55] Zalewski A. 2001 Seasonal and sexual variation in diel activity rhythms of pine marten Martes martes in the Białowieża National Park (Poland). Acta Theriol. **46**, 3. (10.4098/at.arch.01-32)

[56] Zielinski W, Spencer W, Barrett R. 1983 Relationship between food habits and activity patterns of pine martens. J. Mammal. **64**, 387–396.

[57] Yilmaz M, Meister M. 2013 Rapid innate defensive responses of mice to looming visual stimuli. Curr. Biol. **23**, 2011–2015. (10.1016/j.cub.2013.08.015) 24120636 PMC3809337

[58] Sundell J, Dudek D, Klemme I, Koivisto E, Pusenius J, Ylonen H. 2004 Variation in predation risk and vole feeding behaviour: a field test of the risk allocation hypothesis. Oecologia **139**, 157–162. (10.1007/s00442-004-1490-x) 14730443

[59] Bachman GC. 1993 The effect of body condition on the trade-off between vigilance and foraging in belding’s ground squirrels. Anim. Behav. **46**, 233–244. (10.1006/anbe.1993.1185)

[60] Bednarz PA. 2021 Do decibels matter? A review of effects of traffic noise on terrestrial small mammals and bats. Pol. J. Ecol **68**, 323–333. (10.3161/15052249pje2020.68.4.005)

[61] Mazza V, Jacob J, Dammhahn M, Zaccaroni M, Eccard JA. 2019 Individual variation in cognitive style reflects foraging and anti-predator strategies in a small mammal. Sci. Rep **9**, 1–9. (10.1038/s41598-019-46582-1) 31300696 PMC6626059

[62] Lima SL, Bednekoff PA. 1999 Temporal variation in danger drives antipredator behavior: the predation risk allocation hypothesis. Am. Nat. **153**, 649–659. (10.2307/2463621) 29585647

[63] Reilly E. 2024 Red squirrels exhibit antipredator behavioral changes in response to a native predator, the pine marten. Zenodo. (10.5281/zenodo.10600860) PMC12173489 40535941

